# The Effects of Time-Restricted Eating on Glycemic Control, Lipid Profiles, Androgen Levels, and Blood Pressure in PMOS: A Systematic Review and Meta-Analysis

**DOI:** 10.3390/jcm15218350

**Published:** 2026-10-27

**Authors:** Yang Yang, Zheng Liu, Jing Zhang

**Affiliations:** 1 Department of Obstetrics and Gynecology, West China Second University Hospital, Sichuan University, Chengdu 610041, China; yangyang0820@alu.scu.edu.cn (Y.Y.);; 2 Reproductive Endocrinology and Regulation Laboratory, West China Second University Hospital, Sichuan University, Chengdu 610041, China; 3 Key Laboratory of Birth Defects and Related Diseases of Women and Children, Ministry of Education, Sichuan University, Chengdu 610041, China; 4 The Joint Laboratory for Reproductive Medicine of Sichuan University, The Chinese University of Hong Kong, Chengdu 610041, China

**Keywords:** time-restricted eating, polycystic ovary syndrome, polyendocrine metabolic ovarian syndrome, meta-analysis, insulin resistance, hyperandrogenism

## Abstract

**Background/Objectives**: Polyendocrine metabolic ovarian syndrome (PMOS) is a common endocrine–metabolic disorder. Time-restricted eating (TRE) has emerged as a potential dietary intervention, but its efficacy in PMOS remains unclear. This systematic review and meta-analysis aimed to evaluate the effects of TRE on metabolic, hormonal, and anthropometric parameters in women with PMOS. **Methods**: A literature search was conducted using PubMed, Scopus, Web of Science databases, Embase, Cochrane Library, and the WHO International Clinical Trials Registry Platform for studies published from inception to 1 July 2026. Eight studies involving 363 women with PMOS were included in the systematic review. Among these, five controlled studies (four randomised controlled trials and one randomised crossover trial), involving 264 participants, were eligible for quantitative synthesis. **Results**: Quantitative synthesis was conducted using five controlled studies involving 264 participants. Meta-analysis showed that TRE significantly reduced fasting plasma glucose (MD = −0.17 mmol/L, 95% CI: −0.31 to −0.03), fasting insulin (MD = −28.86 pmol/L, 95% CI: −51.98 to −5.74), and HOMA-IR (MD = −0.66, 95% CI: −0.86 to −0.45). TRE also significantly improved QUICKI (SMD = 0.48, 95% CI: 0.05 to 0.92) and reduced HbA1c levels (MD = −1.70%, 95% CI: −3.18 to −0.22). No statistically significant differences were observed for body weight, BMI, waist circumference, lipid profiles (LDL-C, HDL-C, TC, and TG), total testosterone, SHBG, free androgen index, or blood pressure outcomes (SBP P = 0.05; DBP P = 0.11). Subgroup analyses based on comparator type revealed no significant subgroup differences for the key outcomes. **Conclusions**: TRE may improve insulin sensitivity-related outcomes—including fasting plasma glucose, fasting insulin, HOMA-IR, and QUICKI—as well as HbA1c in women with PCOS. However, current evidence does not demonstrate significant effects on anthropometric measures, lipid metabolism, androgen-related outcomes, or blood pressure. Long-term, larger trials are warranted to confirm these benefits.

## 1. Introduction

Polyendocrine metabolic ovarian syndrome (PMOS) is one of the most common endocrine and metabolic disorders affecting women of reproductive age. Approximately 170 million women of reproductive age worldwide are affected by this condition, with a prevalence of approximately 12.5% [1]. In 2026, under the leadership of the International PCOS Network, the condition was officially renamed from ‘polycystic ovary syndrome (PCOS)’ to ‘polyendocrine metabolic ovarian syndrome (PMOS)’, highlighting its underlying metabolic pathophysiology and the multisystemic nature of its associated complications [2]. For many years, patients have commonly presented to obstetrics and gynaecology clinics because of ovarian abnormalities and reproductive dysfunction, while metabolic abnormalities, including insulin resistance, dyslipidaemia, type 2 diabetes, and cardiovascular disease, as well as their associated health risks, have often been overlooked [2,3,4]. PMOS not only has a substantial impact on patients’ quality of life but also imposes a considerable economic burden on healthcare systems worldwide [5].

Lifestyle modification is the first-line treatment for the clinical management of PMOS, with a primary focus on dietary modification and increased physical activity [6]. Time-restricted eating (TRE) is a promising dietary intervention that limits daily energy intake to a predetermined eating window of ≤10 h, followed by a fasting period of at least 14 h. TRE has been shown to confer substantial metabolic benefits in individuals with obesity and type 2 diabetes. Basic research has demonstrated that TRE profoundly influences gene expression in mammals, with nearly 80% of genes showing alterations in expression or circadian rhythms under TRF conditions in at least one tissue [7]. Both animal studies and clinical research have demonstrated that TRE may provide a range of health benefits, including improvements in cardiovascular health, amelioration of metabolic disorders, and reductions in inflammation [8,9,10]. A network meta-analysis of interventions for type 2 diabetes showed that TRE significantly reduced fasting blood glucose, haemoglobin A1c, systolic blood pressure, body weight, body mass index (BMI), and waist circumference [11]. The efficacy of TRE in improving insulin resistance, reducing fasting blood glucose levels, improving lipid profiles, and promoting weight loss has been supported by numerous high-quality studies [12,13,14]. However, research on TRE in the PMOS population remains in its early stages. Some studies have suggested that TRE may improve body weight, insulin resistance, and androgen levels in patients with PMOS [15,16]. However, systematic reviews have indicated that the effects of intermittent fasting on metabolic outcomes and hormonal profiles in patients with PMOS remain unclear [17].

To date, systematic, quantitative, and comprehensive evaluations of the efficacy of TRE interventions in patients with PMOS remain limited. Given the complex and multifactorial nature of PMOS, as well as the lack of satisfactory outcomes from existing dietary management strategies in this population, this study aims to comprehensively evaluate the effects of TRE on metabolic and reproductive endocrine parameters in patients with PMOS through a systematic review and meta-analysis. Furthermore, we aim to quantify the corresponding effect sizes to provide evidence-based guidance for the clinical application of dietary interventions in patients with PMOS.

## 2. Materials and Methods

The present systematic review and meta-analysis was designed, carried out, and documented following the Preferred Reporting Items for Systematic Reviews and Meta-Analyses (PRISMA) 2020 statement [18]. This study protocol has been registered on PROSPERO (CRD420261494810).

### 2.1. The Pico Framework and Inclusion and Exclusion Criteria

#### 2.1.1. The Pico Framework

The inclusion criteria were as follows:

Population: Women diagnosed with PCOS/PMOS according to the Rotterdam criteria or its modified version [19,20].

Intervention: TRE, defined as a daily eating window of ≤10 h lasting at least 4 weeks. Studies implementing TRE alone or TRE combined with calorie restriction were eligible. Studies in which TRE was combined with additional active nutritional interventions (e.g., probiotic supplementation) were excluded unless the additional intervention was applied equally to the comparator group;

Comparison: Free eating or a calorie-restricted diet.

Outcomes: Hormonal parameters, glucose metabolism, lipid profile, blood pressure, and anthropometric outcomes;

#### 2.1.2. Inclusion Criteria

(1) Intervention studies involving adult female participants (aged ≥18 years) diagnosed with PMOS/PCOS according to the Rotterdam criteria. Both controlled trials (including randomised controlled trials and crossover trials) and uncontrolled single-arm intervention studies were eligible for inclusion in the systematic review; (2) trials in which the impact of TRE was assessed on at least one outcome from the following domains: anthropometric indices, metabolic parameters, hormonal markers, and blood pressure; (3) trials providing pre- and post-intervention mean ± standard deviation (SD) values for outcomes meeting the eligibility criteria; and (4) peer-reviewed articles published in English. (5) In the case of multi-arm trials, only the intervention arms that satisfied the eligibility criteria were retained. Specifically, Talebi was a multi-arm trial; only the eTRE plus placebo arm and the DCR plus placebo comparator arm were included in the present review and analyses, whereas the probiotic co-intervention arm was not eligible and was not included [21]. The two publications were treated as reports of the same study, and outcome data were carefully examined to avoid duplicate inclusion [21,22].

Studies with an eligible comparator arm were eligible for quantitative synthesis, whereas single-arm trials were retained for narrative synthesis only and were not pooled.

#### 2.1.3. Exclusion Criteria

Studies were excluded if they met any of the following criteria: (1) studies involving PMOS participants who received TRE in combination with pharmacological treatment (e.g., metformin, oral contraceptives) or surgical intervention; (2) studies combining TRE with additional lifestyle or nutritional interventions that may independently influence metabolic or hormonal outcomes, including structured exercise programmes, probiotic supplementation, or other supplements, were excluded. Studies combining TRE with calorie restriction were eligible because calorie restriction represents a commonly implemented component of dietary TRE interventions; (3) studies with insufficient outcome data for qualitative synthesis; (4) non-peer-reviewed materials, including case reports, narrative reviews, systematic reviews, meta-analyses, conference abstracts, letters or editorials, or those not published in English; (5) studies using animal or in vitro models.

### 2.2. Search Strategy

A comprehensive literature search of the electronic databases PubMed, Scopus, Web of Science, Embase, and Cochrane Library, as well as the WHO International Clinical Trials Registry Platform (ICTRP) was performed up to 1 July 2026. The search targeted human intervention trials examining the effects of TRE in women with PCOS/PMOS, encompassing both controlled trials and single-arm intervention trials. The full search strategy is provided in Table S1.

### 2.3. Study Selection

To ensure accuracy and reproducibility, the search was repeated independently by two authors (Y.Y. and Z.L.). Beyond the database searches, the reference lists of all eligible articles were screened manually to identify additional eligible trials. Any disagreements were settled by discussion; when consensus could not be achieved, a third author (J.Z.) was consulted. The selection process was documented in a PRISMA flowchart, and the reasons for exclusion were recorded at each stage.

### 2.4. Data Extraction

For each eligible trial, the following information was extracted: study identifier, country, sample size, study design, study duration, mean age, baseline BMI, details of the TRE intervention, details of the control diet, and comparator characteristics (where applicable); these data are presented in Table 1. Data extraction was performed independently by two authors (Y.Y. and Z.L.), and any discrepancies were resolved by a third author (J.Z.).

### 2.5. Risk of Bias and Quality of Evidence Assessment

All included RCTs were assessed by two independent reviewers (Y.Y. and Z.L.). The methodological quality of the included RCTs was assessed using the Cochrane Risk of Bias 2 (RoB 2) tool [23]. This tool assesses five potential areas of bias: (1) bias arising from the randomisation process; (2) bias arising from deviation from the intended intervention; (3) bias arising from missing outcome data; (4) bias in the measurement of outcomes; and (5) bias in the selection of outcomes reported. Each domain is categorised as having a “low risk of bias”, “some concerns”, or “high risk of bias”. According to the RoB 2 guidance, if any domain was judged to be at high risk of bias, the overall risk of bias for that study was also judged as high risk.

The certainty of evidence for each outcome was assessed using the Grading of Recommendations Assessment, Development and Evaluation (GRADE) approach. Two reviewers independently evaluated the evidence across five domains: risk of bias, inconsistency, indirectness, imprecision, and publication bias. The results were summarised in a Summary of Findings (SoF) table.”

### 2.6. Statistical Analysis

Meta-analyses were performed with Review Manager (RevMan) version 5.4 (Cochrane Collaboration). Given that pooling required a between-group comparison, only studies with a comparator arm were entered into the quantitative synthesis, whereas data from single-arm intervention trials were described narratively and excluded from the pooled estimates. For each controlled trial contributing to the meta-analysis, we extracted the baseline-to-follow-up change scores together with their standard deviations (SDs) for outcomes meeting the eligibility criteria. Treatment effects were expressed as the mean difference (MD) when studies reported outcomes on a common measurement scale, and as the standardised mean difference (SMD) when the scales differed. For randomised crossover trials, where neither the mean nor the standard deviation (or standard error) of the paired differences has been reported, nor the parametric test statistic required to calculate the standard error of the mean difference, we extracted data from each intervention phase and pooled them using the parallel-groups method [24].

**Table 1:** Characteristics of included studies.

Study	Diagnostic Criteria for PCOS	Country	Design	Sample Size (I/C)	Age (Years)	Baseline BMI (kg/m^2^)	TRE Regimen	Control	Duration	Primary Outcomes	Main Findings
Aminian et al., 2026 [16]	mR	Iran	RCT	25/25	27.7 ± 3.7/27.6 ± 4.1	30.8 ± 2.7/30.3 ± 2.5	eTRE (08:00–18:00)	Ad libitum	6 weeks	FBG, fasting insulin, HOMA-IR, lipids, weight, BMI, WC	↓ FBG, fasting insulin, HOMA-IR, Weight, WC (*p* < 0.05)
Corapi et al., 2026 [15]	R	USA	RCT	26/24	30 ± 6/29 ± 6	35 ± 7/33 ± 9	13:00–19:00 (6 h window)	Usual diet (Total time ≥ 10 h)	6 months	Percentage body weight change	Weight loss; no difference between groups
Feyzioglu et al., 2023 [25]	R	Turkey	Single-arm retrospective	30/—	25.6 ± 2.7	25.1 ± 3.2	13:00–21:00 (8 h window); 16 h fast	—	6 weeks	BMI, WHR, FAI, HOMA-IR, Reproductive hormones, glucose and lipid profile, faecal calprotectin	↓ BMI, WHR, FAI, HOMA-IR, metabolic parameters, faecal calprotectin (all *p* < 0.05)
Floyd et al., 2026 [24]	R	UK	Randomised crossover (4-week washout)	11 (completed)	27 (23–30)	35.0 (30.4–41.4)	6 h self-selected window (e.g., 08:00–14:00)	Ad libitum	12 weeks	HbA1c, weight, BMI, hip and waist circumference	↓ HbA1c, Weight, BMI, hip and waist circumference vs. ad libitum
Jakubowicz et al., 2013 [26]	R	Israel	RCT	25/26	31.3 ± 0.8/32.6 ± 0.7	23.8 ± 0.2/23.6 ± 0.2	High-calorie breakfast + low-calorie dinner (Total time ≤ 9 h per day)	Low-calorie breakfast + high-calorie dinner (Total time ≤ 9 h per day)	12 weeks	Glucose AUC, insulin AUC, free testosterone, SHBG, 17OHP, ovulation rate	BF group: ↓ glucose AUC 7%, ↓ insulin AUC 54%, ↓ free testosterone 50%, ↑ SHBG 105%, ↑ ovulation rate; D group: no change
Talebi et al., 2024, Talebi et al., 2025 * [21,22]	R	Iran	RCT	30/30	30.2 ± 5.6/31.1 ± 5.8	29.8 ± 3.6/30.7 ± 3.5	14:10 eTRE (08:00–18:00) ± placebo	DCR (25% energy restriction) + placebo	8 weeks	Blood pressure, CRP, TAC, blood pressure	All groups ↓ CRP (*p* < 0.005); TAC improved in eTRE + probiotic and DCR; no between-group differences
Li et al., 2021 [27]	R	China	Single-arm prospective	18/—	18–31	29.75 ± 4.31	08:00–21:00 (8 h window); 16 h fast	—	5 weeks	BMI, WHR, FAI, HOMA-IR, Reproductive hormones, glucose and lipid profile, faecal calprotectin	↓ BMI, body fat, TT, FAI, HOMA-IR, ALT, hsCRP, improved menstrual regularity (73.3%); ↑ SHBG (all (*p* < 0.05)
Zheng et al., 2025 [28]	R	China	RCT	46/47	26.1 ± 4.8/27.2 ± 5.6	30.7 ± 3.9/30.2 ± 4.1	8 h window + 1200–1500 kcal/day	DCR (1200–1500 kcal/day)	16 weeks	Weight, HOMA-IR	TRE −7.47 kg vs. DCR −4.38 kg (*p* < 0.05); HOMA-IR decreased in both groups

R, Rotterdam criteria; mR, modified Rotterdam criteria; DCR, Daily Calorie Restriction; Single-arm studies were included in the systematic review but were not included in quantitative synthesis. * The two articles are based on the same study but report different outcome measures.

When baseline-to-follow-up changes in means and standard deviations were unavailable in the primary reports, we estimated these values with established approaches. For studies reporting the median together with the first and third quartiles (Q1, Q3), the corresponding mean and standard deviation were obtained through the conversion procedure proposed by Wan et al. [29]. Because matched-pair data were statistically insufficient, the crossover trial was analysed using a parallel-group comparison based on the data that were available [30]. Between-study heterogeneity was measured with the I^2^ statistic and graded as low (0–30%), moderate (30–60%), substantial (60–90%), or considerable (>90%). A random-effects model based on the DerSimonian and Laird method was applied. A two-tailed *p*-value below 0.05 was taken to indicate statistical significance.

Subgroup analyses were undertaken by comparator type, since distinct control conditions address distinct clinical questions. Studies were assigned to one of two subgroups: (1) TRE versus ad libitum/free eating, and (2) TRE versus a calorie-restricted diet. For each subgroup, pooled effects were estimated separately. Between-subgroup differences were examined with the test for subgroup differences. Where adequate controlled comparisons existed, comparator-based subgroup analyses were carried out for key metabolic and anthropometric outcomes, namely body weight, waist circumference, and HOMA-IR.

## 3. Results

### 3.1. Literature Selection

**Figure 1:**
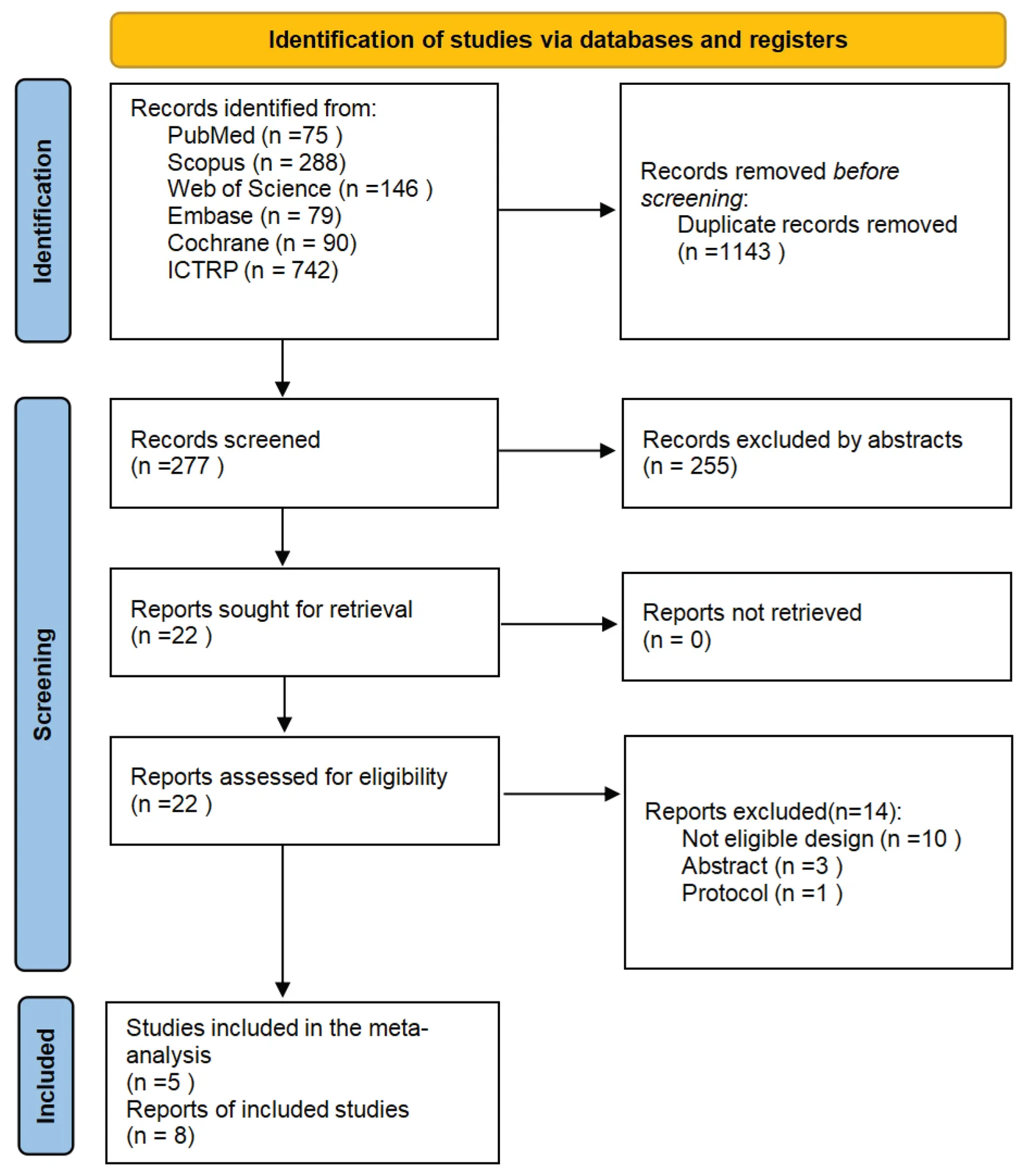
PRISMA flow diagram.

The updated search identified a total of 1420 records from five electronic databases and one clinical trial registry (Figure 1). After removing duplicates, 277 records remained for title and abstract screening. Following screening, 255 records were excluded because they did not meet the eligibility criteria, and 22 reports were assessed through full-text review. Ultimately, eight studies were included in the systematic review. Among these, five were randomised controlled trials, one was a randomised crossover trial, and two were uncontrolled single-arm intervention studies. Five controlled studies (four RCTs and one crossover trial) were included in the primary meta-analysis, whereas the two single-arm studies and the study by Jakubowicz et al., which evaluated meal timing within a restricted eating window rather than TRE compared with an unrestricted eating pattern, were synthesised narratively only [26]. The Floyd study employed a randomised crossover design, comprising 12 weeks of TRE, a 4-week washout period, and 12 weeks of ad libitum feeding. The original paper did not formally model period, sequence, or carry-over effects; the authors explicitly stated that no such formal modelling was undertaken, citing the short duration of the washout period, the likely minimal biological carry-over effects, and the exploratory nature of the study with its limited sample size [24]. The characteristics of the included studies are shown in Table 1.

This systematic review included a total of eight studies, comprising 363 participants. These studies consisted of five randomised controlled trials, one randomised crossover trial, two single-arm intervention studies, and one meal-timing intervention study with a restricted eating window. The included studies were published between 2013 and 2026 and originated from six countries: Iran, the United States, Turkey, the United Kingdom, Israel, and China.

The sample sizes of the individual studies ranged from 11 to 93 participants. The mean age of the participants was 25.6–32.6 years, with a baseline BMI of 23.6–35.0 kg/m^2^. With the exception of the study by Jakubowicz et al., which included leaner women with PCOS (mean BMI approximately 23.7 kg/m^2^), the participants in the remaining studies were predominantly overweight or obese.

The intervention programmes varied in terms of eating window duration and timing. Three studies employed a morning time-restricted eating regimen with an approximately 10 h eating window (08:00–18:00) [16,21,22]. Feyzioglu reported an 8 h eating window (13:00–21:00) [25], whereas Zheng applied an 8 h eating window combined with a prescribed energy intake of 1200–1500 kcal/day [28]. Two studies used a 6 h eating window: one with a fixed window of 13:00–19:00 [15], and another allowing participants to select their own 6 h window [24]. Li investigated a 16:8 time-restricted feeding intervention in women with PCOS, with participants consuming food within an 8 h window (08:00–21:00) and fasting for approximately 16 h [27]. It is worth noting that Jakubowicz et al. adopted a chrononutrition-based intervention, in which participants consumed meals within a total eating window of ≤9 h, while the primary difference between groups was the distribution of caloric intake between breakfast and dinner [26]. The intervention duration ranged from 5 weeks to 6 months.

The control groups also differed: two studies employed ad libitum feeding [16,24]; and Aminian’s study was a three-arm parallel randomised controlled trial, which included only the eTRE group and the ad libitum control group [16]; one used usual dietary habits [15]; two used calorie-restricted control diets [21,28]; and one compared a large breakfast with reverse meal distribution (a small breakfast and a large dinner) [26]. The two single-arm studies were not included in quantitative synthesis [25,27]. Talebi et al. reported two publications from the same research group using a similar intervention framework; outcome data were carefully examined to avoid duplicate inclusion [21,22].

### 3.2. Meta-Analysis Outcomes

#### 3.2.1. The Effect of TRE on Anthropometric Indices

**Figure 2:**
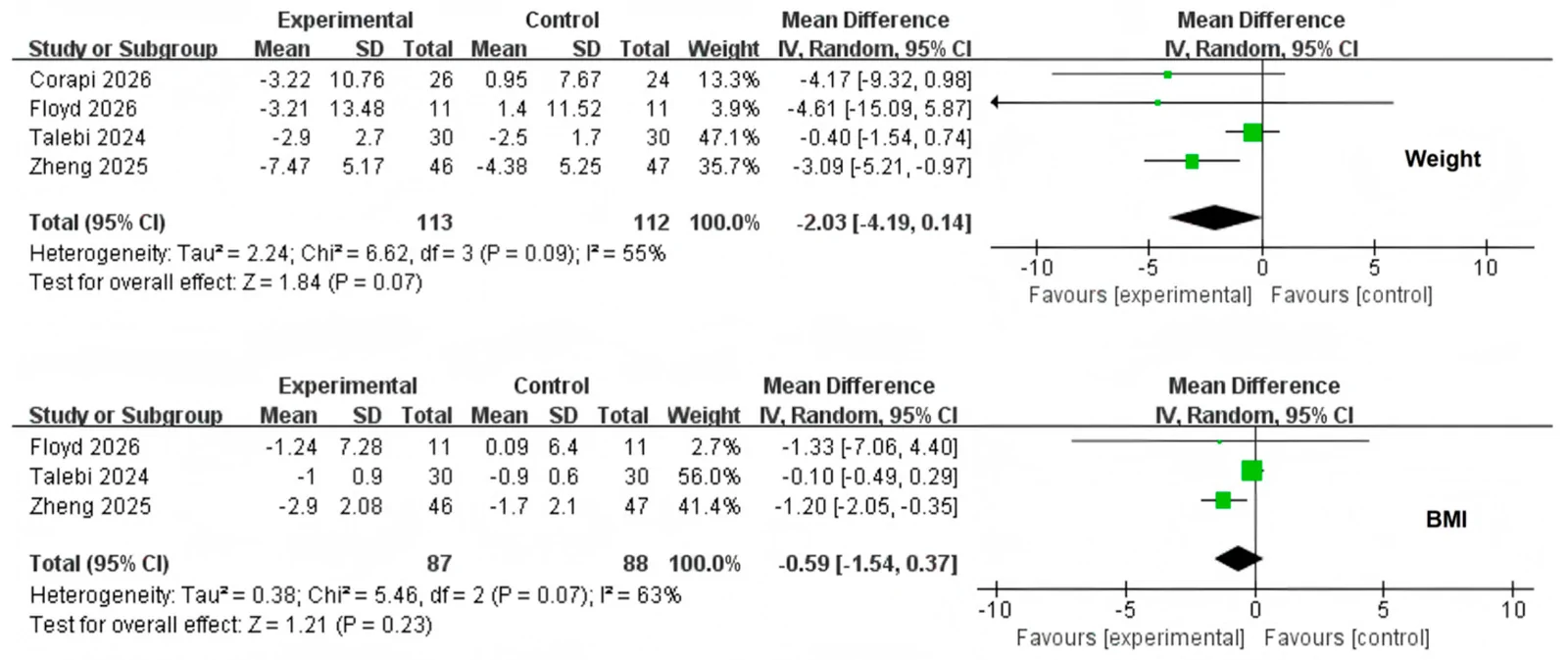
Forest plot showing body weight (W), body mass index (BMI), and waist circumference (WC) in patients with PMOS/PCOS following the TRE diet versus the control diet.

A total of four studies assessed the effect of time-restricted eating (TRE) on body weight. Analysis using a random-effects model showed that there was no statistically significant difference in body weight (MD = −2.03 kg, 95% CI: −4.19, 0.14, *p* = 0.07, I^2^ = 55%) and BMI (MD = −0.59 kg/m^2^, 95% CI: −1.54, 0.37, *p* = 0.23, I^2^ = 63%) (Figure 2).

#### 3.2.2. Effects of TRE on Glucose Metabolism

**Figure 3:**
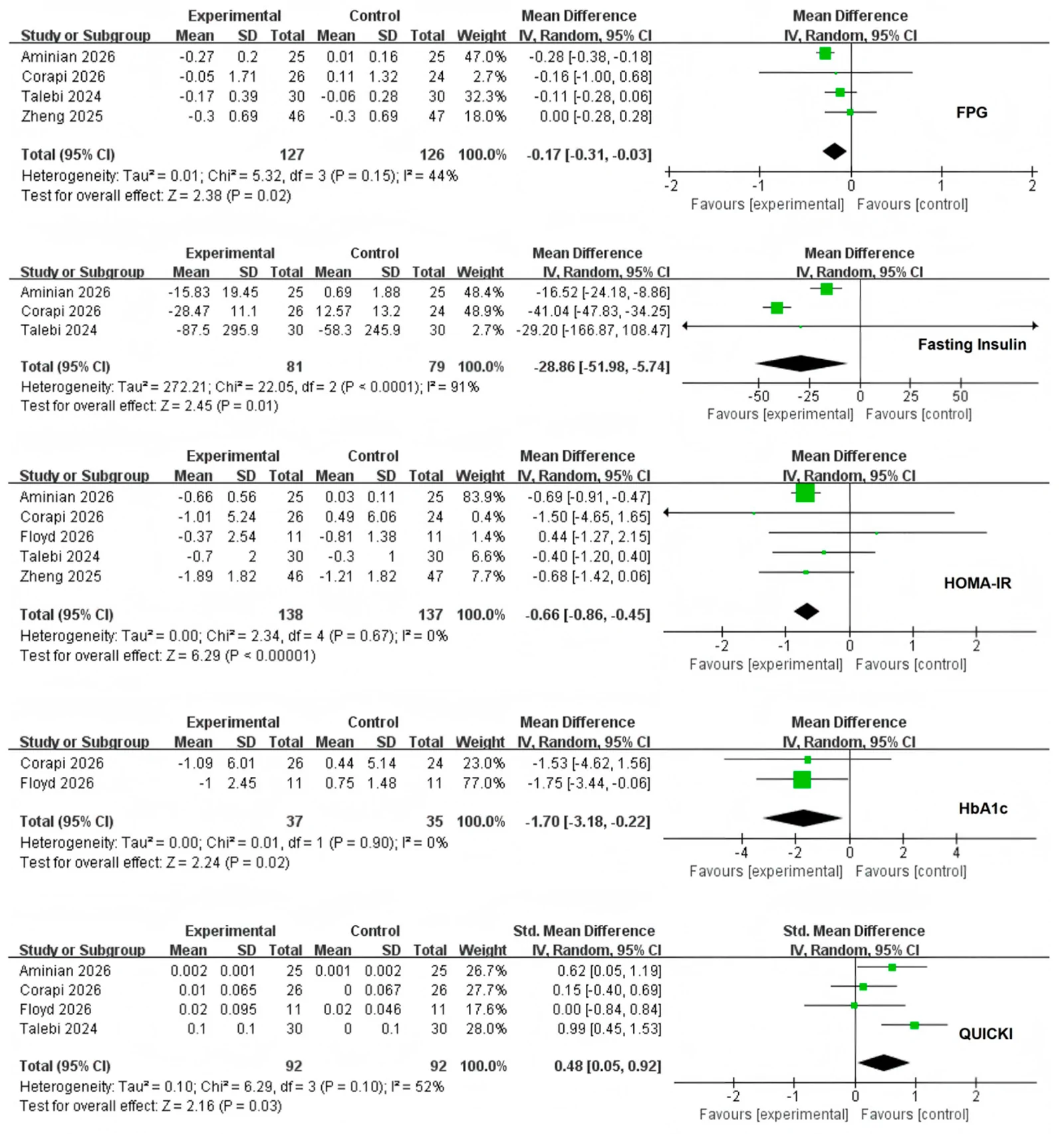
Forest plots comparing the TRE diet with the control diet in patients with PMOS/PCOS in terms of FPG, fasting insulin, HOMA-IR, HbA1c, and QUCIK.

Analysis using a random-effects model showed that TRE significantly reduced fasting plasma glucose (FPG) (MD = −0.17 mmol/L, 95% CI: −0.31, −0.03, *p* = 0.02, I^2^ = 44%), fasting insulin (MD = −28.86 pmol/L, 95% CI: −51.98, −5.74, *p* = 0.01, I^2^ = 91%), and HOMA-IR (MD = −0.66, 95% CI: −0.86, −0.45, *p* < 0.00001, I^2^ = 0%). TRE also significantly improved QUICKI (SMD = 0.48, 95% CI: 0.05, 0.92, *p* = 0.03, I^2^ = 52%). Two studies examined HbA1c, and TRE significantly reduced HbA1c compared with the control group (MD = −1.70%, 95% CI: −3.18, −0.22, *p* = 0.02, I^2^ = 0%) (Figure 3).

#### 3.2.3. Effects of TRE on Lipid Metabolism

**Figure 4:**
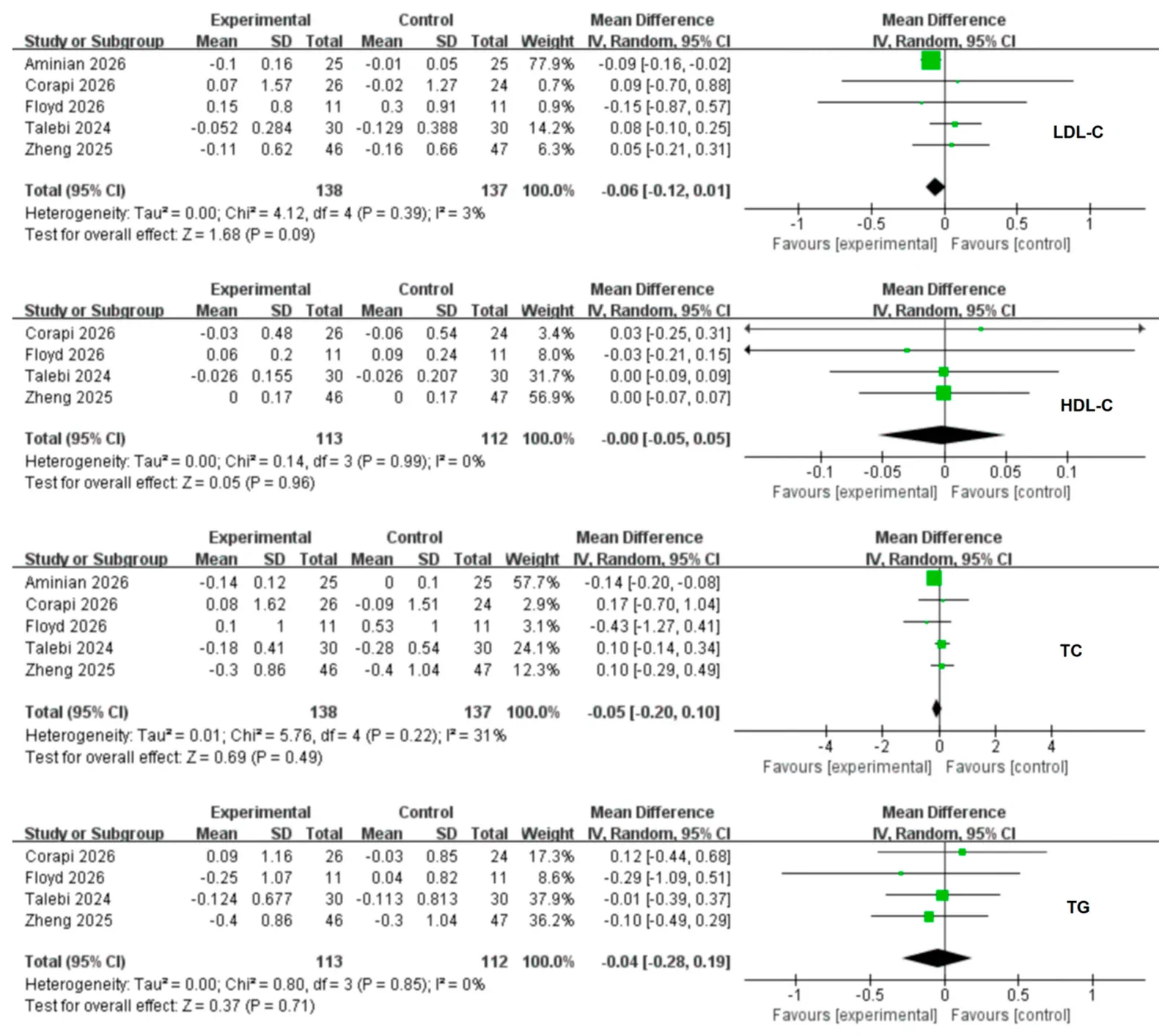
Forest plots showing LDL-C, HDL-C, TC, and TG levels in patients with PMOS/PCOS following the TRE diet versus the control diet.

Five studies were included, and no statistically significant differences were found between the TRE and control groups in terms of LDL-C (*p* = 0.09), HDL-C (*p* = 0.96), TC (*p* = 0.49), or TG (*p* = 0.71) (Figure 4).

#### 3.2.4. Effects of TRE on Androgens

**Figure 5:**
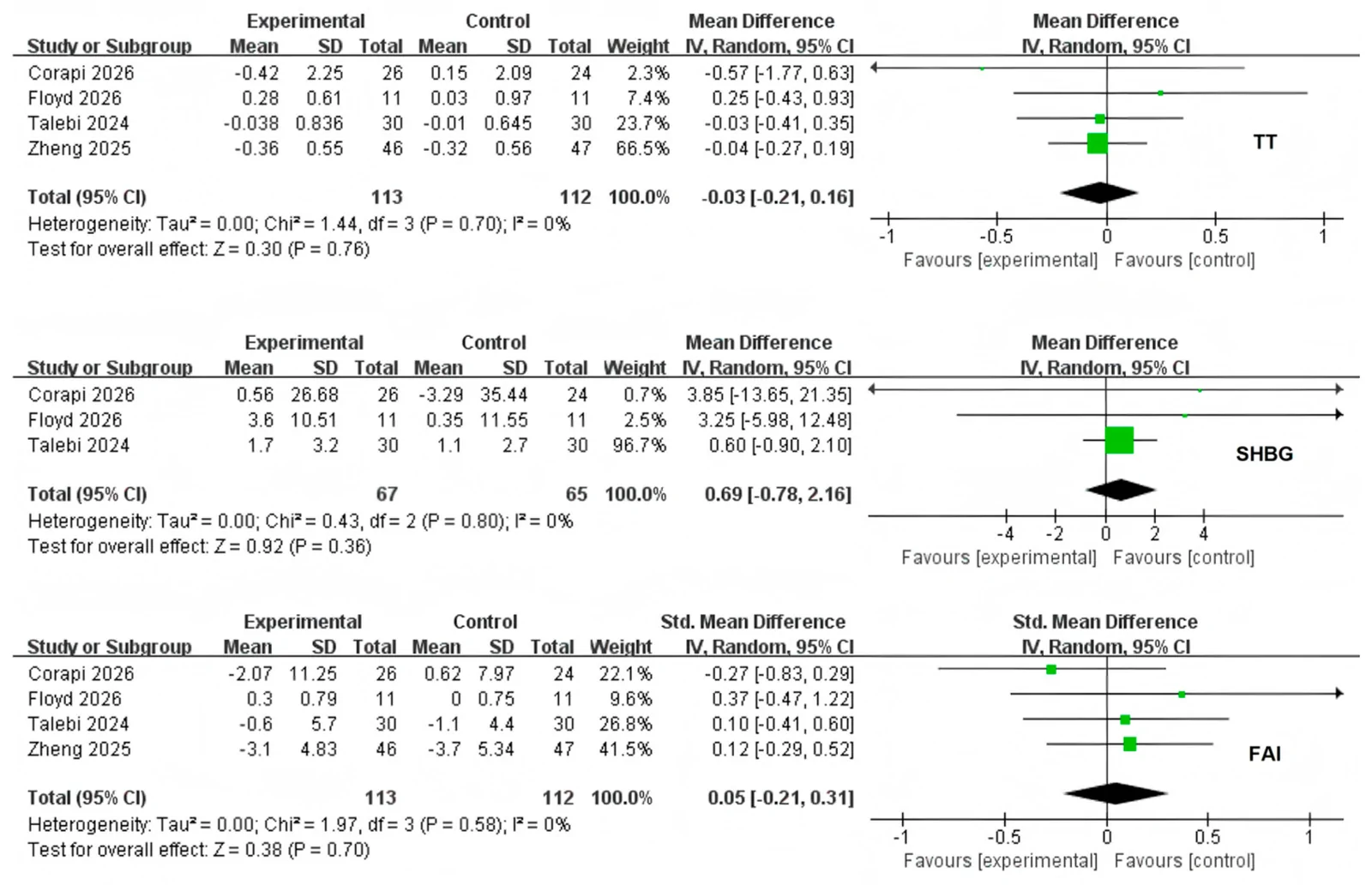
Forest plot comparing TT, SHBG, and FAI levels in patients with PMOS/PCOS following the TRE diet versus the control diet.

Four studies assessed the effects of TRE on androgens; there was no statistically significant difference in SHBG levels (*p* = 0.36), total testosterone (*p* = 0.76), or the FAI (*p* = 0.70) (Figure 5) between the TRE group and the control group.

#### 3.2.5. Effects of TRE on Sbp and Dbp

**Figure 6:**
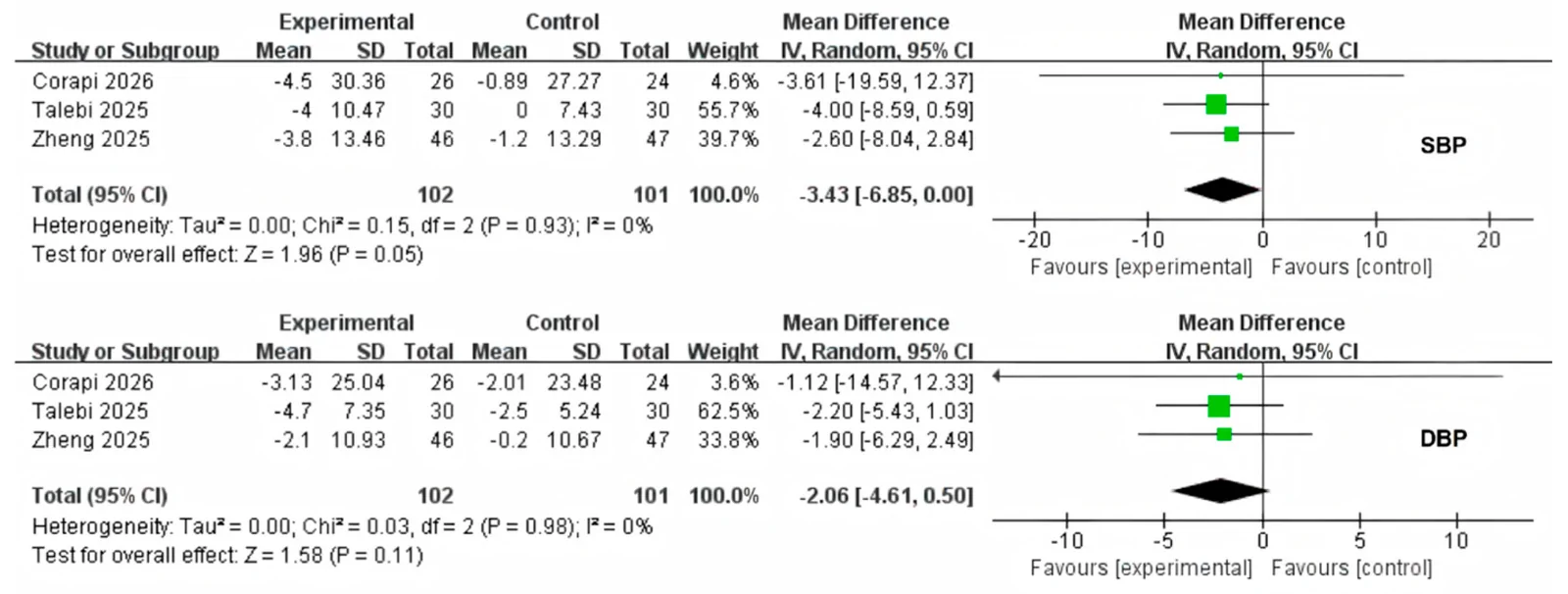
Forest plot showing the effects of the TRE diet versus the control diet on SBP and DBP in patients with PMOS/PCOS.

A total of three studies examined the effects of TRE on blood pressure; the analysis revealed no statistically significant differences between the TRE group and the control group in terms of SBP (*p* = 0.05) and DBP (*p* = 0.11) (Figure 6).

### 3.3. Subgroup Analyses and Sensitivity Analysis

**Figure 7:**
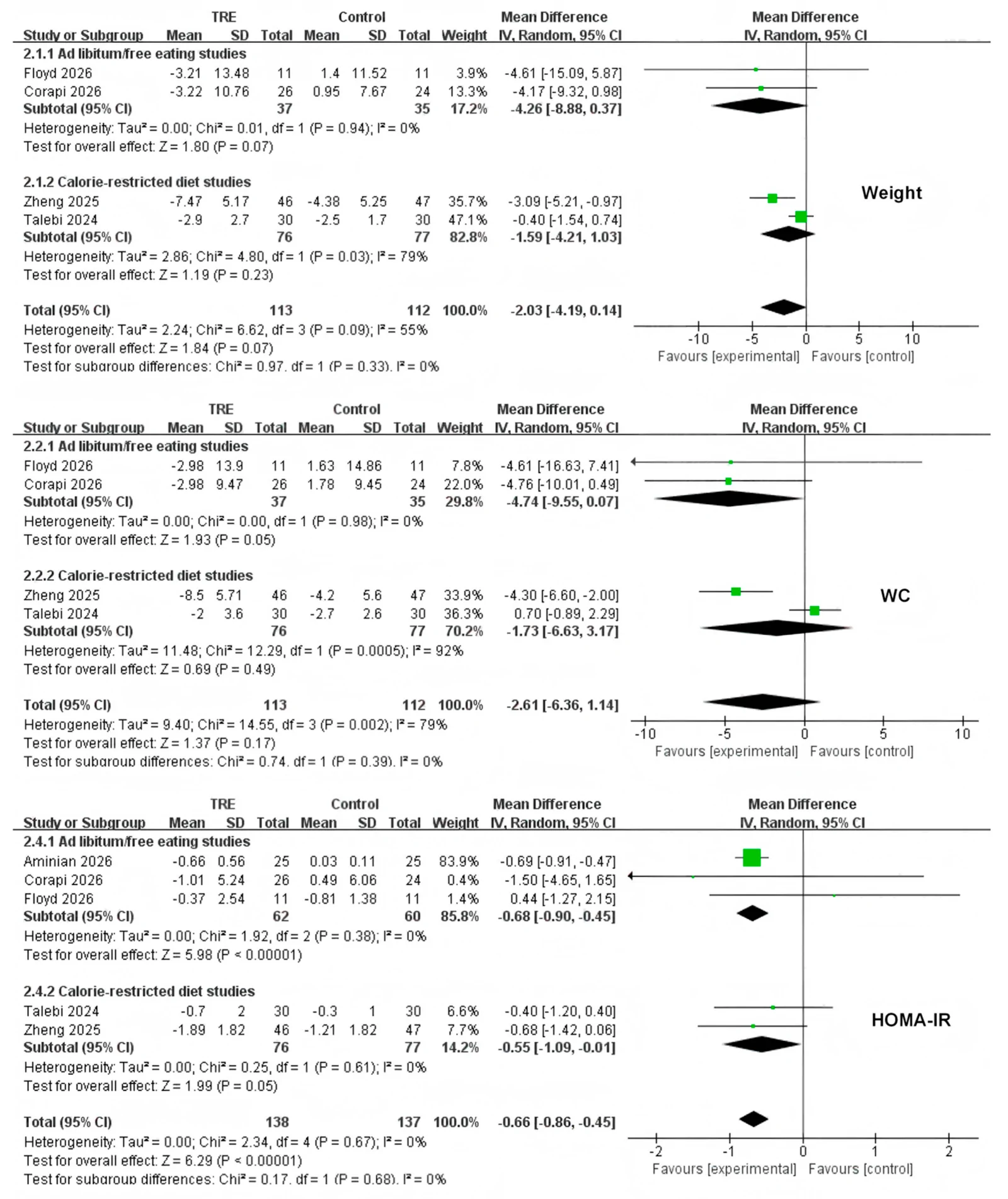
Forest plots of subgroup analyses stratified by comparator type for body weight, waist circumference, and HOMA-IR.

Given the potential influence of energy restriction on metabolic outcomes, comparator-based subgroup analyses were performed for key anthropometric and metabolic outcomes. Subgroup analyses were conducted according to comparator type to explore whether the effects of TRE differed between studies comparing TRE with ad libitum eating and those comparing TRE with calorie-restricted diets. For body weight, TRE was associated with a non-significant reduction in body weight compared with both ad libitum eating (MD −4.26 kg, 95% CI −8.88 to 0.37; *p* = 0.07) and calorie-restricted diets (MD −1.59 kg, 95% CI −4.21 to 1.03; *p* = 0.23). No statistically significant difference was observed between the two comparator subgroups (*p* for subgroup difference = 0.33), suggesting that comparator type did not significantly modify the effect of TRE on body weight. For waist circumference, TRE showed a trend toward greater reduction compared with ad libitum eating (MD −4.74 cm, 95% CI −9.55 to 0.07; *p* = 0.05), whereas the effect was not significant when compared with calorie-restricted diets (MD −1.73 cm, 95% CI −6.63 to 3.17; *p* = 0.49). However, the difference between comparator subgroups was not statistically significant (*p* for subgroup difference = 0.39). For HOMA-IR, TRE significantly reduced insulin resistance compared with ad libitum eating (MD −0.69, 95% CI −0.90 to −0.47; *p* < 0.00001) and also showed a borderline significant reduction compared with calorie-restricted diets (MD −0.55, 95% CI −1.09 to −0.01; *p* = 0.05). Nevertheless, no significant subgroup difference was detected (*p* for subgroup difference = 0.68), indicating that the magnitude of the effect did not differ significantly according to comparator type (Figure 7).

To assess the robustness of the pooled estimates, a ‘leave-one-out’ sensitivity analysis was conducted for all outcome measures (Table S2). A stepwise exclusion method was employed to perform sensitivity analyses for all outcomes. HOMA-IR was the only outcome that remained statistically significant across all iterations, indicating a robust beneficial effect of TRE on insulin resistance. In contrast, the effects on body weight, BMI, waist circumference, fasting glucose, fasting insulin, SHBG, QUICKI, and HbA1c were influenced by the exclusion of individual studies and should therefore be interpreted cautiously. The pooled estimates for lipid profiles (TC, TG, LDL-C, and HDL-C), total testosterone, FAI, and blood pressure outcomes (SBP and DBP) remained non-significant across sensitivity analyses, indicating consistent absence of evidence for these outcomes.

### 3.4. Narrative Synthesis of Studies Not Included in the Meta-Analysis and Menstrual Outcomes

Three studies were not included in the quantitative synthesis due to methodological or intervention-related considerations. Two uncontrolled single-arm studies evaluated the effects of TRE in women with PCOS. Li et al. investigated a 5-week 16:8 TRE intervention in women with anovulatory PCOS and reported significant improvements in anthropometric parameters, insulin resistance indices, androgen-related markers, SHBG levels, and menstrual regularity [27]. Similarly, Feyzioglu et al. evaluated a 6-week 8 h TRE intervention and observed reductions in BMI, waist-to-hip ratio, FAI, HOMA-IR, and metabolic parameters [25]. However, because both studies lacked comparator groups, their findings should be interpreted cautiously, and causal effects of TRE cannot be established.

The study by Jakubowicz et al. was not included in the primary meta-analysis because both intervention groups consumed food within a restricted eating window (≤9 h), with the main difference being the distribution of energy intake between breakfast and dinner rather than TRE compared with an unrestricted eating pattern [26]. This study reported that a high-calorie breakfast and low-calorie dinner pattern resulted in improvements in glucose and insulin responses, free testosterone, SHBG levels, and ovulation rate compared with the reverse meal distribution pattern. These findings may reflect the combined effects of meal timing and circadian-aligned energy distribution rather than the isolated effects of TRE.

Talebi’s study found that there were no significant differences in menstrual patterns between the three groups—eTRE combined with probiotics, eTRE combined with a placebo, and daily calorie restriction. Among these, the proportions of participants experiencing improved menstrual regularity were 33.3 per cent and 36.7 per cent in the eTRE combined with placebo group and the daily calorie restriction group, respectively, and this improvement paralleled weight loss [21].

### 3.5. Risk of Bias Assessment

**Figure 8:**
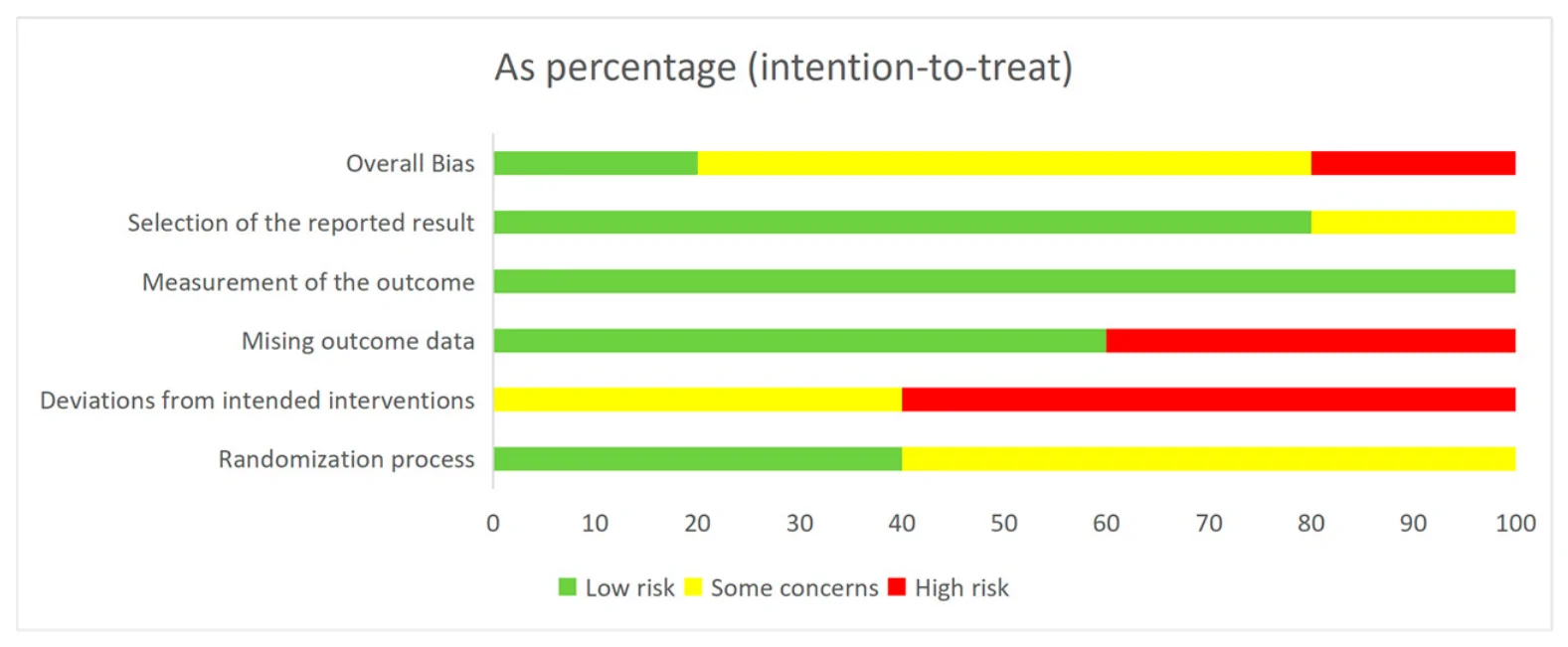
Bias risk percentage chart.

**Figure 9:**
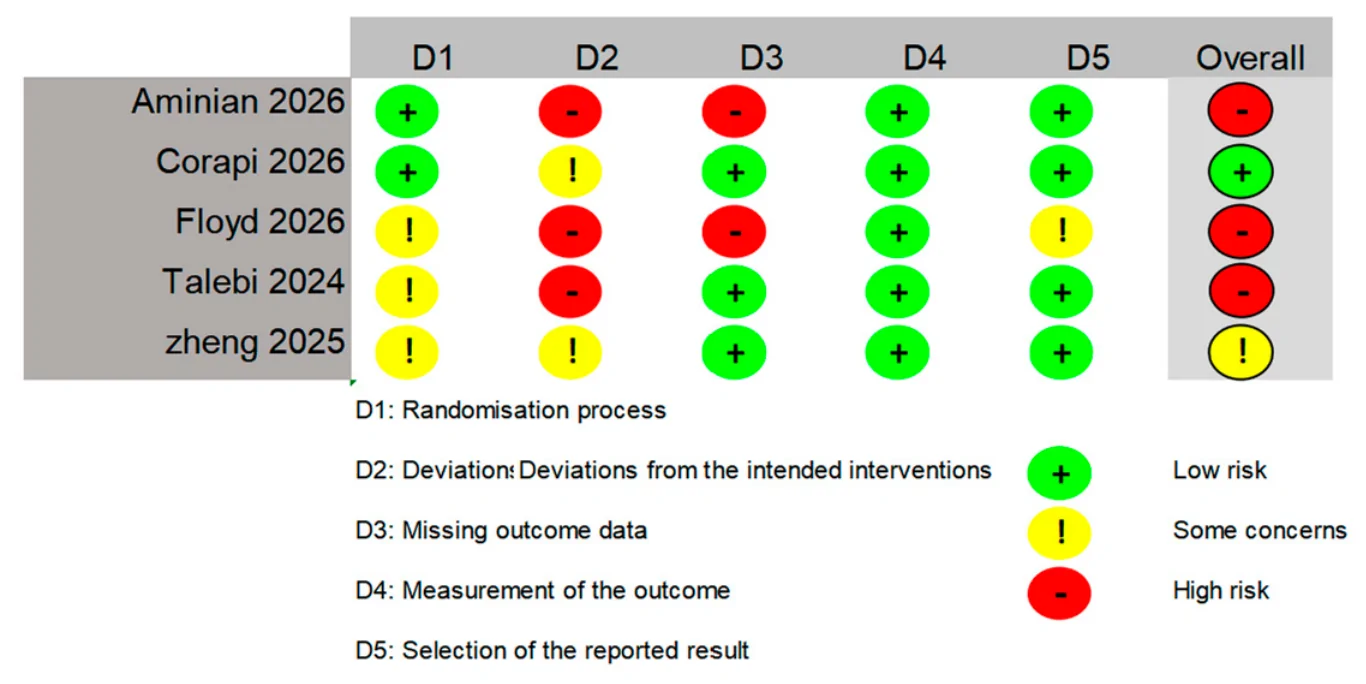
Risk of bias of the included studies.

A risk of bias assessment was conducted for all five included randomised controlled trials using the Cochrane Risk of Bias 2.0 tool. According to the final assessment (Figure 8 and Figure 9), one study was judged to be at low risk [15], one study raised some concerns [28], and three studies were judged to be at high risk [16,21,24].

In most trials, adequate randomisation procedures were reported, with 40% of studies rated as low risk and 60% as some concerns in the randomisation process (D1). The domain of “deviations from intended interventions” (D2) represented the most significant source of bias, with 60% of studies rated as high risk and the remaining 40% as some concerns. This is expected given the inherent difficulty of blinding participants and personnel in dietary behaviour interventions. Furthermore, 40% of studies were rated as high risk for “missing outcome data” (D3), whereas 60% were rated as low risk. Notably, the domain of “measurement of the outcome” (D4) was rated as low risk across all studies, as the primary outcomes (e.g., FPG, fasting insulin, HOMA-IR) were derived from objective laboratory measurements. Finally, 80% of studies were rated as low risk for the “selection of the reported result” (D5), reflecting the presence of prospective registration, while 20% raised some concerns.

### 3.6. Quality of Evidence Assessment

The GRADE assessment of the certainty of evidence is presented in Table 2. The certainty of evidence ranged from moderate to very low. Specifically, the evidence was rated as moderate for FPG, HOMA-IR, and lipid profiles, and low to very low for fasting insulin, HbA1c, body weight, BMI, waist circumference, androgen outcomes, and blood pressure. The primary reasons for downgrading included risk of bias (lack of blinding in dietary interventions) and inconsistency (high heterogeneity).

## 4. Discussion

This systematic review comprehensively evaluated the effects of TRE as an intervention in women with PMOS/PCOS. The main findings are as follows: TRE significantly reduced FPG, fasting insulin levels, HOMA-IR, and HbA1c, while improving the QUICKI. However, TRE did not result in significant improvements in body weight, BMI, waist circumference, LDL-C, HDL-C, TC, TG, TT, SHBG, FAI, SBP, or DBP. These findings suggest that the metabolic benefits of TRE in women with PMOS/PCOS may be primarily driven by improvements in glucose homeostasis and insulin sensitivity, rather than anthropometric measures, lipid profiles, androgen regulation, or blood pressure outcomes. Our findings are partly consistent with those of previous analyses and build upon them. Compared with the meta-analysis by Aolymat et al. [17], which searched only three databases and included four trials, our study conducted a more extensive search. Whilst Aolymat reported that intermittent fasting had no significant effect on fasting blood glucose and insulin, our study demonstrated significant improvements in fasting insulin, HOMA-IR and QUICKI [17]. This discrepancy may be primarily attributable to heterogeneity in the interventions: Aolymat combined TRE with other IF regimens for analysis as a generalised concept. Our positive findings regarding improvements in insulin sensitivity are consistent with the conclusions of Hamsho [31].

With regard to body weight and anthropometric measures, our meta-analysis did not demonstrate statistically significant effects of TRE on body weight (MD = −2.03 kg, 95% CI: −4.19 to 0.14, *p* = 0.07), BMI (MD = −0.59 kg/m^2^, 95% CI: −1.54 to 0.37, *p* = 0.23), or waist circumference (*p* = 0.17). Although the pooled estimate for body weight favoured TRE, the confidence interval crossed the null, and this finding should therefore be interpreted as a non-significant trend rather than a confirmed benefit. These findings differ from some previous trials. A randomised controlled trial involving adults with obesity demonstrated that both 4 h and 6 h TRE were effective in reducing body weight, with weight loss reaching its greatest magnitude at approximately three months of intervention [32]. Another 8-week TRE study also reported a significant reduction in body weight [33]. These previous trials were conducted largely in adults with obesity or type 2 diabetes rather than in women with PCOS, which may partly explain the discrepancy. The follow-up periods in the studies included in our analysis ranged from 6 weeks to 6 months, and the pooled sample size and between-study heterogeneity may have limited our ability to detect significant anthropometric effects. Notably, the weight-loss effects of TRE may not depend solely on deliberate calorie counting; instead, restricting the daily eating window to 4–10 h, corresponding to a fasting period of 14–20 h, may naturally reduce daily energy intake [32,33,34]. Furthermore, TRE does not appear to compromise dietary macronutrient composition; in a 3-month intervention involving a 10 h TRE regimen, the proportion of energy derived from macronutrients remained unchanged in adults with T2DM [35]. This relative simplicity, which does not require intentional calorie restriction, may facilitate long-term adherence and potentially support sustained metabolic improvements, particularly among individuals with obesity [36]. International evidence-based guidelines emphasise lifestyle modification as the first-line approach for improving insulin resistance, metabolic health, and reproductive outcomes in women with PCOS [6,20]. Moderate weight loss of 5–10% has been associated with improvements in clinical manifestations of PCOS, including restoration of ovulation, increased pregnancy rates, and reductions in insulin and androgen levels [37]. However, no significant improvement in waist circumference was observed in our study, which may be related to the characteristic abdominal adiposity phenotype observed in women with PCOS and the possibility that the intervention duration was insufficient to induce detectable changes in fat distribution.

With regard to glucose metabolism, this study found that TRE significantly reduced FPG, fasting insulin, and HOMA-IR and improved QUICKI; moreover, HbA1c was also significantly reduced. These findings are broadly consistent with previous studies reporting that TRE reduces fasting insulin and HOMA-IR [38]. However, our observation of a significant reduction in FPG differs from the findings of Sutton et al., who reported that FPG remained largely unchanged after a 5-week eTRF intervention in men with prediabetes [38]; Sutton et al. enrolled men with prediabetes. Similarly, HbA1c—which reflects average blood glucose over the preceding 2–3 months and typically requires at least three months of intervention to demonstrate meaningful changes [39]—was significantly reduced in our analysis. It should be noted that Lowe et al. studied a mixed population of men and women with a mean age of 46.5 years. Given that our HbA1c finding was based on a limited number of studies, it should be interpreted with caution. Furthermore, a recent meta-analysis indicated that early time-restricted eating (eTRE) can significantly reduce FPG and HOMA-IR, although the effect sizes were relatively small (MD −3.64 mg/dL and −0.42, respectively), suggesting that the glycaemic effects of TRE may depend on the specific intervention protocol [40]. These findings provide further evidence supporting a potential independent role of TRF in improving insulin sensitivity.

With regard to blood lipids and blood pressure, this study found that TRE had no significant effects on LDL-C, HDL-C, TC, TG, SBP, or DBP. This finding is consistent with previous reviews, which have suggested that the effects of TRE on plasma lipid levels are generally limited, whereas its effects on blood pressure are more heterogeneous [36]. With regard to blood pressure, a cross-sectional meta-analysis of community-dwelling adults found no consistent association between TRE, defined as a feeding window of ≤12 h, and cardiometabolic health [41], whereas another 8-week study in adults with overweight or obesity found that changes in serum metabolites were significantly associated with reductions in SBP [42], suggesting that time-restricted feeding may contribute to improvements in cardiovascular and metabolic health through modulation of the gut microbiota and circulating metabolites. The absence of significant effects in our study may therefore be partly attributable to the relatively mild baseline abnormalities in lipid and blood pressure levels among women with PCOS or to the possibility that the follow-up period was insufficient for measurable cardiovascular benefits to emerge.

With regard to androgens and SHBG, this study observed no significant changes in SHBG, testosterone, or FAI. A previous systematic review of RCTs involving predominantly obese women similarly found that TRE did not have consistent effects on testosterone, oestrogen, progesterone, LH, cortisol, DHEA, or SHBG [43]; whereas non-randomised studies in women with PCOS have reported reductions in testosterone and LH, although these findings may have been confounded by weight loss. An 8-week TRE study using a 4–6 h eating window in premenopausal women found that androgen and SHBG levels remained unchanged, whereas DHEA levels decreased [44]. In contrast, a recent systematic review indicated that TRF interventions can significantly reduce the free androgen index and increase SHBG in women with PCOS, while also improving menstrual regularity, with 33–40% of participants experiencing normalisation of their menstrual cycles, accompanied by reductions in total testosterone, FAI, AMH, and LH [45,46]. Our meta-analysis did not demonstrate significant changes in SHBG, testosterone, or FAI. This discrepancy may be attributable to the relatively small sample sizes of the included studies, phenotypic heterogeneity among women with PCOS, and differences in TRE protocols.

The metabolic benefits of TRE may involve multiple biological pathways. Circadian rhythm disruption is considered an important contributing factor to the pathogenesis and clinical manifestations of PCOS [47]. By enhancing circadian signalling, TRE may restore the circadian oscillations of short-chain fatty acid (SCFA)-producing gut microbiota, with rhythmic SCFAs acting as ‘metabolic timekeepers’ to resynchronise autophagy rhythms in peripheral tissues, such as the heart, thereby improving mitochondrial function [48]. Emerging evidence suggests that gut microbiota dysbiosis may contribute to the pathogenesis of PCOS by altering intestinal permeability, promoting endotoxaemia, and exacerbating insulin resistance and hormonal disturbances [49]. Hyperandrogenism, a key feature of PCOS, may itself play a critical role in shaping the microbial community [50]. Modification of the gut microbiota through probiotics, prebiotics, and synbiotics has therefore been proposed as a potential therapeutic strategy for PCOS [49,50]. These circadian and microbial alterations may converge on the insulin–SHBG–androgen axis. Theoretically, by improving insulin sensitivity, TRE could attenuate the hyperinsulinaemia-induced suppression of hepatic SHBG production, thereby increasing SHBG levels and reducing free androgen levels. In the present study, however, TRE did not significantly alter SHBG or androgen levels despite improvements in insulin sensitivity. Further long-term studies with larger sample sizes are warranted to clarify this discrepancy.

The studies included in this review employed eating windows ranging from 6 to 10 h, with differences observed in the efficacy of individual regimens. Early TRE (eTRE) appeared to confer greater benefits than midday or late TRE in reducing fasting blood glucose and HOMA-IR [40]. In contrast, an eating window of ≤8 h per day was significantly associated with an increased risk of obesity (BMI ≥30) [51]. However, more restrictive daily TRE regimens may adversely affect mood and dietary satisfaction, whereas intermittent TRF (iTRF) may similarly reproduce beneficial effects in advanced metabolic diseases while offering better tolerability [52,53]. A study in prediabetic mice showed that a 10 h TRF window significantly alleviated features of cardiac ageing; however, this protective effect was attenuated during the diabetic stage, suggesting that disease severity may influence the efficacy of TRE [48]. In women with PCOS, although short-term 16:8 TRE over 4 weeks was feasible and well tolerated, no specific improvements in cognitive function were observed [54]. Therefore, individualised selection of the timing and duration of the eating window, together with consideration of patient adherence and tolerability, will be important for the successful clinical translation of TRE.

## 5. Strengths and Limitations

Building on previous systematic reviews, this review updates the evidence regarding time-restricted eating interventions in women with PMOS/PCOS. Compared with an earlier meta-analysis [31], this study further incorporates the most recent research data to date on TRE interventions in this population. Notably, this study is the first to incorporate blood pressure measurements into a quantitative meta-analysis and to conduct subgroup analyses based on control group type (free feeding versus calorie restriction) to explore the potential impact of energy restriction on metabolic outcomes. Furthermore, we performed sensitivity analyses using the leave-one-out method and rigorously graded the certainty of evidence for each outcome using the GRADE tool, thereby comprehensively assessing the robustness of the pooled results.

Despite the rigorous methodological approach described above, this study has some limitations. Firstly, there were significant variations in the methodological quality of the included studies; due to the inherent nature of dietary behaviour interventions, it is extremely difficult to blind participants and implementers, resulting in a high risk of bias, which may affect the estimates of the pooled effects. Secondly, owing to the limited number of studies assessing individual outcomes, we were unable to assess publication bias using funnel plots and Egger’s test. Thirdly, some outcomes had small sample sizes and high heterogeneity, resulting in imprecise results. Sensitivity analyses indicated that the TRE’s improvement in HOMA-IR was the most stable and robust finding, whilst the statistical significance of other favourable outcomes (such as body weight and waist circumference) was more susceptible to the exclusion of individual studies. Consistent with this, the GRADE assessment revealed that the certainty of the evidence for the various outcomes ranged from moderate to very low, with the primary reasons for downgrading being risk of bias, inconsistency and imprecision. Consequently, the metabolic benefits of TRE for women with PMOS/PCOS still require confirmation through large-scale, long-term, methodologically rigorous randomised controlled trials.

## 6. Conclusions

This systematic review and meta-analysis suggest that TRE may have favourable effects on glycaemic control and insulin sensitivity-related outcomes in women with PMOS, as indicated by significant reductions in fasting plasma glucose, fasting insulin, HOMA-IR, and HbA1c, and an improvement in QUICKI. However, no statistically significant effects were observed on body weight, BMI, waist circumference, lipid profiles, androgen-related outcomes, or blood pressure. Given the limited number of included studies, relatively small sample sizes, and short follow-up periods, these findings should be interpreted as preliminary and hypothesis-generating rather than definitive. Future randomised controlled trials with larger sample sizes, longer follow-up, and adequate statistical power are needed to determine whether TRE confers long-term metabolic and reproductive health benefits in women with PMOS. Future studies should pre-specify and report reproductive outcomes, such as menstrual regularity, ovulation, and pregnancy, using standardised definitions to facilitate pooling in future meta-analyses. In the present review, reproductive outcomes could not be pooled because of insufficient and inconsistently reported data and were therefore synthesised narratively.

## Figures and Tables

**Table 2:** GRADEpro evaluation of the evidence produced.

Certainty Assessment						No. of Patients		Effect	Degree of General Certainty of Evidence
No. of Studies	Study Design	Risk of Bias	Inconsistency	Indirect Evidence	Inaccuracy	Other Considerations	No. of Patients (TRE/Control)	Effect (MD/SMD, 95% CI)	
Fasting plasma glucose (FPG)									
4	Randomised controlled trials	Serious ^1^	Not serious	Not serious	Not serious	None	27/126	MD −0.17 mmol/L (−0.31 to −0.03)	⊕⊕⊕○ Moderate
Fasting insulin									
3	Randomised controlled trials	Serious ^1^	Very serious ^2^	Not serious	Not serious	None	81/79	MD −20.41 pmol/L (−37.05 to −3.78)	⊕⊕○○ Low
HOMA-IR									
4	Randomised controlled trials	Serious ^1^	Not serious	Not serious	Not serious	None	127/126	MD −0.67 (−0.88 to −0.47)	⊕⊕⊕○ Moderate
QUICKI									
4	Randomised controlled trials	Serious ^1^	Serious ^3^	Not serious	Not serious	None	92/92	SMD 0.48 (0.05 to 0.92)	⊕⊕○○ Low
HbA1c									
2	Randomised controlled trials	Serious ^1^	Not serious	Not serious	Very serious ^4^	None	37/35	MD −1.70% (−3.18 to −0.22)	⊕⊕○○ Low
Body weight									
4	Randomised controlled trials	Serious ^1^	Not serious	Not serious	Serious ^5^	None	113/112	MD −2.03 kg (−4.19 to 0.14)	⊕⊕○○ Low
BMI									
3	Randomised controlled trials	Serious ^1^	Serious ^6^	Not serious	Serious ^5^	None	87/88	MD −0.59 kg/m^2^ (−1.54 to 0.37)	⊕○○○ Very Low
Waist circumference									
4	Randomised controlled trials	Serious ^1^	Not serious	Not serious	Serious ^5^	None	113/112	MD −2.61 cm (−6.63 to 3.17)	⊕⊕○○ Low
Lipid profiles (LDL-C, HDL-C, TC, TG)									
5	Randomised controlled trials	Serious ^1^	Not serious	Not serious	Not serious	None	138/137	Not significant (*p* > 0.05 for all)	⊕⊕⊕○ Moderate
Androgen outcomes (TT, SHBG, FAI)									
4	Randomised controlled trials	Serious ^1^	Not serious	Not serious	Not serious	None	113/112	Not significant (*p* > 0.05 for all)	⊕⊕⊕○ Moderate
Blood pressure (SBP, DBP)									
3	Randomised controlled trials	Serious ^1^	Not serious	Not serious	Serious ^5^	None	102/101	SBP *p* = 0.05; DBP *p* = 0.11	⊕⊕○○ Low

^1^ Risk of Bias (Serious): Downgraded by 1 level due to the inability to blind participants and personnel in dietary interventions, leading to potential performance bias. ^2^ Inconsistency (Very serious): Downgraded by 1 level due to high statistical heterogeneity (I^2^ = 88%). ^3^ Inconsistency (Serious): Downgraded by 1 level due to moderate statistical heterogeneity (I^2^ = 52%). ^4^ Inaccuracy (Very serious): Downgraded by 1 level because only 2 studies were included, with a wide confidence interval, resulting in limited precision. ^5^ Inaccuracy (Serious): Downgraded by 1 level because the 95% confidence interval crosses the null effect (*p* > 0.05), indicating imprecise results. ^6^ Inconsistency (Serious): Downgraded by 1 level due to moderate statistical heterogeneity (I^2^ = 63%). GRADE Certainty Levels: ⊕⊕⊕○ Moderate; ⊕⊕○○ Low; ⊕○○○ Very Low.

## Data Availability

The data extraction dataset generated and analysed during this study are openly available in Figshare at https://doi.org/10.6084/m9.figshare.34562091 (accessed on 16 October 2026). No new primary data were generated in this study.

## Supplementary Materials

The following supporting information can be downloaded at: https://www.mdpi.com/article/10.3390/jcm15218350/s1, Table S1: Database Search strategy, Table S2: leave-one-out’ sensitivity analysis, Table S3: PRISMA 2020 checklist [18].

## Abbreviations

The following abbreviations are used in this manuscript:

AMH	Anti-Müllerian hormone
BMI	Body mass index
DBP	Diastolic blood pressure
DHEA	Dehydroepiandrosterone
eTRE	Early time-restricted eating
FAI	Free androgen index
HbA1c	Glycated haemoglobin
HIIT	High-intensity interval training
HOMA-IR	Homeostatic model assessment of insulin resistance
iTRF	Intermittent time-restricted feeding
LH	Luteinising hormone
LDL-C	Low-density lipoprotein cholesterol
NAD	Nicotinamide adenine dinucleotide
PCOS	Polycystic ovary syndrome
PMOS	Polyendocrine metabolic ovarian syndrome
QUICKI	Quantitative insulin sensitivity check index
RCT	Randomised controlled trial
SBP	Systolic blood pressure
SCFA	Short-chain fatty acid
SHBG	Sex hormone-binding globulin
T2DM	Type 2 diabetes mellitus
TC	Total cholesterol
TG	Triglycerides
TRE	Time-restricted eating
