# Supplementary material for: The Effects of Time-Restricted Eating on Glycemic Control, Lipid Profiles, Androgen Levels, and Blood Pressure in PMOS: A Systematic Review and Meta-Analysis
Source: J Clin Med. 2026 Oct 27;15(21):8350. doi: 10.3390/jcm15218350 (PMC13763992; doi:10.3390/jcm15218350)
Supplement: Supplementary file 1 [file jcm-15-08350-s001.zip › Table S3 PRISMA 2020 checklist.pdf]

PRISMA 2020 Checklist

Section and Topic	Item #	Checklist item	Location where item is reported
**TITLE**			
Title	1	Identify the report as a systematic review.	Line 2-4/Page 1
**ABSTRACT**			
Abstract	2	See the PRISMA 2020 for Abstracts checklist.	Another list
**INTRODUCTION**			
Rationale	3	Describe the rationale for the review in the context of existing knowledge.	Line 40-75/page 2
Objectives	4	Provide an explicit statement of the objective(s) or question(s) the review addresses.	Line 75-85/page 2
**METHODS**			
Eligibility criteria	5	Specify the inclusion and exclusion criteria for the review and how studies were grouped for the syntheses.	Line 101-116/page 3
Information sources	6	Specify all databases, registers, websites, organisations, reference lists and other sources searched or consulted to identify studies. Specify the date when each source was last searched or consulted.	Line 118-121/page 3
Search strategy	7	Present the full search strategies for all databases, registers and websites, including any filters and limits used.	Line 121/page 3
Selection process	8	Specify the methods used to decide whether a study met the inclusion criteria of the review, including how many reviewers screened each record and each report retrieved, whether they worked independently, and if applicable, details of automation tools used in the process.	Line 123-128/page 3
Data collection process	9	Specify the methods used to collect data from reports, including how many reviewers collected data from each report, whether they worked independently, any processes for obtaining or confirming data from study investigators, and if applicable, details of automation tools used in the process.	Line 130-134/page 3
Data items	10a	List and define all outcomes for which data were sought. Specify whether all results that were compatible with each outcome domain in each study were sought (e.g. for all measures, time points, analyses), and if not, the methods used to decide which results to collect.	Line 99-100/page 3
	10b	List and define all other variables for which data were sought (e.g. participant and intervention characteristics, funding sources). Describe any assumptions made about any missing or unclear information.	Line 170/page 4
Study risk of bias assessment	11	Specify the methods used to assess risk of bias in the included studies, including details of the tool(s) used, how many reviewers assessed each study and whether they worked independently, and if applicable, details of automation tools used in the process.	Line 145-159/page 4
Effect measures	12	Specify for each outcome the effect measure(s) (e.g. risk ratio, mean difference) used in the synthesis or presentation of results.	Line 144-148/page 4
Synthesis methods	13a	Describe the processes used to decide which studies were eligible for each synthesis (e.g. tabulating the study intervention characteristics and comparing against the planned groups for each synthesis (item #5)).	Line94-100/page 4
	13b	Describe any methods required to prepare the data for presentation or synthesis, such as handling of missing summary statistics, or data conversions.	Line 149-159/page 4
	13c	Describe any methods used to tabulate or visually display results of individual studies and syntheses.	Line 144-148/page 4
	13d	Describe any methods used to synthesize results and provide a rationale for the choice(s). If meta-analysis was performed, describe the model(s), method(s) to identify the presence and extent of statistical heterogeneity, and software package(s) used.	Line 145-159/page 4
	13e	Describe any methods used to explore possible causes of heterogeneity among study results (e.g. subgroup analysis, meta-regression).	Line 145-159/page 4
	13f	Describe any sensitivity analyses conducted to assess robustness of the synthesized results.	Line288-300/page11
Reporting bias assessment	14	Describe any methods used to assess risk of bias due to missing results in a synthesis (arising from reporting biases).	Line 302-315/page 11
Certainty assessment	15	Describe any methods used to assess certainty (or confidence) in the body of evidence for an outcome.	Line 302-315/page 11

PRISMA

PRISMA 2020 Checklist

Section and Topic	Item #	Checklist item	Location where item is reported
**RESULTS**			
Study selection	16a	Describe the results of the search and selection process, from the number of records identified in the search to the number of studies included in the review, ideally using a flow diagram.	Line 163-170/page4
	16b	Cite studies that might appear to meet the inclusion criteria, but which were excluded, and explain why they were excluded.	Line 163-170/page4
Study characteristics	17	Cite each included study and present its characteristics.	Line 177-197/page4
Risk of bias in studies	18	Present assessments of risk of bias for each included study.	Line 302-315/page11
Results of individual studies	19	For all outcomes, present, for each study: (a) summary statistics for each group (where appropriate) and (b) an effect estimate and its precision (e.g. confidence/credible interval), ideally using structured tables or plots.	Line 232-300/page8-11
Results of syntheses	20a	For each synthesis, briefly summarise the characteristics and risk of bias among contributing studies.	Line 232-300/page8-11
	20b	Present results of all statistical syntheses conducted. If meta-analysis was done, present for each the summary estimate and its precision (e.g. confidence/credible interval) and measures of statistical heterogeneity. If comparing groups, describe the direction of the effect.	Line 232-300/page8-11
	20c	Present results of all investigations of possible causes of heterogeneity among study results.	No application
	20d	Present results of all sensitivity analyses conducted to assess the robustness of the synthesized results.	Line288-300/[page 11
Reporting biases	21	Present assessments of risk of bias due to missing results (arising from reporting biases) for each synthesis assessed.	Line 302/page 11
Certainty of evidence	22	Present assessments of certainty (or confidence) in the body of evidence for each outcome assessed.	Line 302/page 11
**DISCUSSION**			
Discussion	23a	Provide a general interpretation of the results in the context of other evidence.	Line 324-451/page 13-15
	23b	Discuss any limitations of the evidence included in the review.	Line 470-487/page 15-16
	23c	Discuss any limitations of the review processes used.	Line 470-487/page 15-16
	23d	Discuss implications of the results for practice, policy, and future research.	Line 489-499/page16
**OTHER INFORMATION**			
Registration and protocol	24a	Provide registration information for the review, including register name and registration number, or state that the review was not registered.	Line 90/page 3
	24b	Indicate where the review protocol can be accessed, or state that a protocol was not prepared.	Line 90/page 3
	24c	Describe and explain any amendments to information provided at registration or in the protocol.	No application
Support	25	Describe sources of financial or non-financial support for the review, and the role of the funders or sponsors in the review.	Line 506/page16
Competing interests	26	Declare any competing interests of review authors.	Line 510/page16

PRISMA 2020 Checklist

Section and Topic	Item #	Checklist item	Location where item is reported
Availability of data, code and other materials	27	Report which of the following are publicly available and where they can be found: template data collection forms; data extracted from included studies; data used for all analyses; analytic code; any other materials used in the review.	Line 508/page16

From: Page, M.J.; McKenzie, J.E.; Bossuyt, P.M.; Boutron, I.; Hoffmann, T.C.; Mulrow, C.D.; Shamseer, L.; Tetzlaff, J.M.; Akl, E.A.; Brennan, S.E.; et al. The PRISMA 2020 statement: An updated guideline for reporting systematic reviews. *Syst. Rev.* **2021**, *10*, 89. https://doi.org/10.1186/s13643-021-01626-4. This work is licensed under CC BY 4.0. To view a copy of this license, visit https://creativecommons.org/licenses/by/4.0/
